# The Formation of D-Allulose 3-Epimerase Hybrid Nanoflowers and Co-Immobilization on Resins for Improved Enzyme Activity, Stability, and Processability

**DOI:** 10.3390/ijms25126361

**Published:** 2024-06-08

**Authors:** Wentao Ding, Chensa Liu, Chi Huang, Xin Zhang, Xinyi Chi, Tong Wang, Qingbin Guo, Changlu Wang

**Affiliations:** 1 School of Food Science and Engineering, Tianjin University of Science and Technology, Tianjin 300457, China; ding_wt@tust.edu.cn (W.D.); 21844917lcs@mail.tust.edu.cn (C.L.); hc99@mail.tust.edu.cn (C.H.); zhangxin991124@mail.tust.edu.cn (X.Z.); 21845934cxy@mail.tust.edu.cn (X.C.); 22142126@mail.tust.edu.cn (T.W.); 2 State Key Laboratory of Food Nutrition and Safety, Tianjin University of Science and Technology, Tianjin 300457, China

**Keywords:** organic–inorganic hybrid nanoflower, D-allulose 3-epimerase, resin, immobilization

## Abstract

As a low-calorie sugar, D-allulose is produced from D-fructose catalyzed by D-allulose 3-epimerase (DAE). Here, to improve the catalytic activity, stability, and processability of DAE, we reported a novel method by forming organic–inorganic hybrid nanoflowers (NF-DAEs) and co-immobilizing them on resins to form composites (Re-NF-DAEs). NF-DAEs were prepared by combining DAE with metal ions (Co^2+^, Cu^2+^, Zn^2+^, Ca^2+^, Ni^2+^, Fe^2+^, and Fe^3+^) in PBS buffer, and were analyzed by scanning electron microscopy (SEM), Fourier transform infrared spectroscopy, and X-ray diffraction. All of the NF-DAEs showed higher catalytic activities than free DAE, and the NF-DAE with Ni^2+^ (NF-DAE-Ni) reached the highest relative activity of 218%. The NF-DAEs improved the thermal stability of DAE, and the longest half-life reached 228 min for NF-DAE-Co compared with 105 min for the free DAE at 55 °C. To further improve the recycling performance of the NF-DAEs in practical applications, we combined resins and NF-DAEs to form Re-NF-DAEs. Resins and NF-DAEs co-effected the performance of the composites, and ReA (LXTE-606 neutral hydrophobic epoxy-based polypropylene macroreticular resins)-based composites (ReA-NF-DAEs) exhibited outstanding relative activities, thermal stabilities, storage stabilities, and processabilities. The ReA-NF-DAEs were able to be reused to catalyze the conversion from D-fructose to D-allulose, and kept more than 60% of their activities after eight cycles.

## 1. Introduction

D-allulose 3-epimerase (DAE) catalyzes the isomerization of the hydroxyl group at the C-3 position of D-fructose, and is the key enzyme for the industrial production of D-allulose, an emerging low-calorie [1] monosaccharide with a sucrose flavor, good safety [2,3], and an anti-obesity function [4,5,6]. However, free DAEs have several drawbacks that limit their industrial application and increase production costs, such as the low reactivity and thermal stability in reaction conditions [3]. One possible solution is using an immobilized enzyme, which combines the enzyme with an immobilized carrier and improves the catalytic activity and stability [7], as well as the enzyme processing performance [8].

He W et al. immobilized DAEs on the surface of bacillus subtilis spores, and the immobilized DAEs retained more than 60% of their activity after five continuous reaction cycles [9]. Samir et al. immobilized DAEs on graphene oxide, and the immobilized DAEs retained more than 50% of their activity after five continuous reaction cycles [7]. Xin G et al. immobilized DAEs by a Resin-SpyCatcher/SpyTag-DAE system, and the immobilized DAEs showed higher thermal and pH stabilities compared with the control [8]. Those immobilization methods showed advantages in improving the stability and processability of the DAEs, but most of the immobilizations reduced the catalytic activity of the enzyme [10], which may lead to an increase in enzyme usage.

Forming organic–inorganic hybrid nanoflowers (NFs) is a simple, carrier-free, and low-energy-cost method for enzyme immobilization [11,12,13,14,15]. Zare et al. [16] combined Cu^2+^ with the enzyme to form hybrid nanoflowers, and by using similar approaches, other metal ions, such as Co^2+^ [17], Zn^2+^ [18], Ca^2+^ [13,15], Ni^2+^ [19], Fe^3+^ [20,21], and Fe^2+^ [22], were also reported to form NFs with proteins and increased catalytic activities [23]. The structural morphologies, catalytic properties, stability, and reusability of NFs are closely related to the type of enzyme and metal ions, the ratio of enzymes to metal ions, as well as the synthesis conditions [24,25]. In a very recent study, Xin et al. reported a customized self-assembled protein–bimetallic hybrid nanoflower system, and found that most of the resulting NFs decreased the catalytic activities, except for the NF-DAE-Co/Mn [23]. As immobilized enzyme modifiers, nanoflowers could modify the enzyme’s morphological properties to produce a more biocompatible shape and release more accessible surface activities, and thus enhance the catalytic activity and stability [26]. The hybrid nanoflowers are scaled from several hundred nanometers to tens of micrometers [17], which enables the efficient interaction between substrates and enzymes. However, in our search, we found nanoflowers are difficult to separate from a reaction regent with high-throughput centrifugation, and cannot be easily reused in industrial applications. Thus, finding an ideal immobilizing method that meets the high catalytic activity, thermal stability, and processing performance simultaneously is still a considerable challenge [27].

In order to address this challenge and improve the application performance of DAEs, we prepared DAE hybrid nanoflowers (NF-DAEs) with seven different metal ions, and systematically investigated the structural morphology, catalytic activity, and thermal stability of those NF-DAEs. Additionally, we found a drawback of the NFs in the reusability due to the bad separation from the solvent with a high viscosity and density through centrifugation. We also report on a novel strategy that overcame this by preparing resin–nanoflower composites, which provide new insights into the application of nanoflowers.

## 2. Results and Discussion

### 2.1. Preparation and Characterizations of NF-DAEs

**Figure 1:**
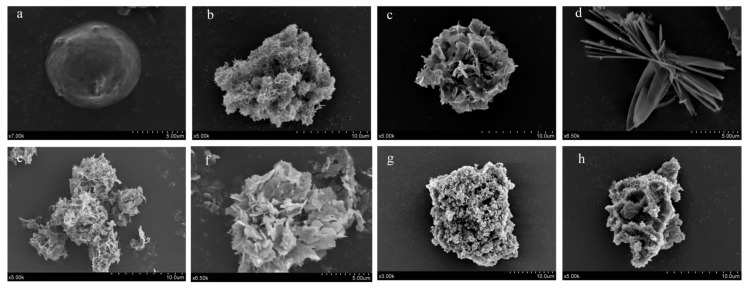
SEM images of free DAE and NF-DAEs. (**a**) Free DAE (freeze-dried), (**b**) NF-DAE-Co, (**c**) NF-DAE-Cu, (**d**) NF-DAE-Zn, (**e**) NF-DAE-Ca, (**f**) NF-DAE-Ni, (**g**) NF-DAE-Fe(ii), and (**h**) NF-DAE-Fe(iii). Magnifications are in the lower-left corner of the image.

To prepare the organic–inorganic hybrid nanoflowers with D-allulose 3-epimerase (DAE), we first expressed and purified the DAE as the organic component, and formed hybrid materials by co-precipitating the DAE with seven well-documented phosphates, including Co^2+^, Cu^2+^, Zn^2+^, Ca^2+^, Ni^2+^, Fe^2+^, and Fe^3+^. These precipitations (hypothetical hybrid nanoflowers) were named NF-DAE-Co, NF-DAE-Cu, NF-DAE-Zn, NF-DAE-Ca, NF-DAE-Fe(ii), and NF-DAE-Fe(iii), respectively, and were characterized by scanning electron microscopy (SEM). The SEM photos of seven NF-DAEs and free enzymes (freeze-dried) are shown in Figure 1. Except for the free DAE (Figure 1a), all of the seven metal phosphates formed ordered crystal structures with the DAE (Figure 1b–h). The NF-DAE-Co, NF-DAE-Cu, NF-DAE-Zn, NF-DAE-Ca, and NF-DAE-Ni (the NF-DAE with Co^2+^, Cu^2+^, Zn^2+^, Ca^2+^, and Ni^2+^, respectively) formed petal-like microstructures with multilayered lamellar surfaces (Figure 1b–f). NF-DAE-Fe(ii) and NF-DAE-Fe(iii) (the NF-DAE with Fe^2+^ and Fe^3+^) formed nanoflowers formed by tiny spherical structures (Figure 1g,h). With SEM observation, we confirmed that the sizes of the NF-DAE-Co, NF-DAE-Cu, NF-DAE-Zn, NF-DAE-Ca, NF-DAE-Fe(ii), and NF-DAE-Fe(iii) were 75–130, 120–125, 83–130, 25–83, 134–153, 169–192, and 92–124 μm, respectively. The microstructures of those NF-DAEs (Figure 1b–h) were different with precipitations of metal phosphates without DAE (Figure S1, Additional File), indicating that the flower-like microstructure of the NF-DAE results from the interaction between protein and metal phosphate [23].

**Figure 2:**
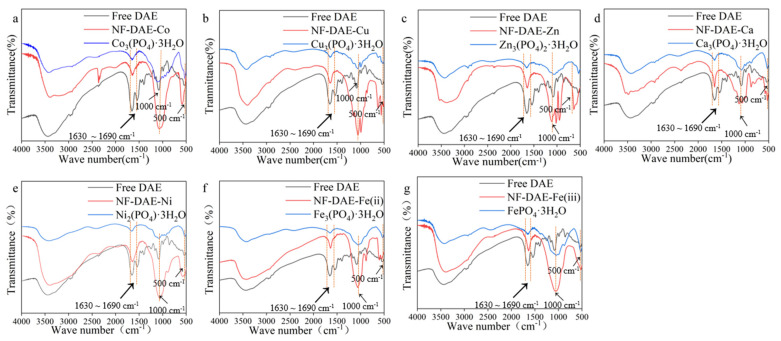
FTIR spectra of free DAE (black), NF-DAEs (red), and metal phosphates (blue). (**a**–**g**) show the FTIR spectra of the NF-DAE-Co, NF-DAE-Cu, NF-DAE-Zn, NF-DAE-Ca, NF-DAE-Ni, NF-DAE-Fe(ii), and NF-DAE-Fe(iii), respectively, and the comparison with that of the free DAE and mental phosphate.

The structures of the NF-DAEs were further studied by FTIR spectra analysis, and the results are shown in Figure 2. The typical absorption peaks of the free DAE (black line) are 1630 cm^−1^~1690 cm^−1^, which was contributed to by the amide bond in the protein. On the other hand, the typical absorption peaks of the metal phosphate were at 500 cm^−1^ and 1000 cm^−1^ (blue line), which were contributed to by the P=O and P-O bonds, respectively [28]. Meanwhile, the FTIR spectra of the NF-DAEs (red line) showed that all of the absorptions were at 500 cm^−1^, 1000 cm^−1^, and 1630 cm^−1^~1690 cm^−1^, indicating that the NF-DAEs contained either DAE or metal phosphate components.

**Figure 3:**
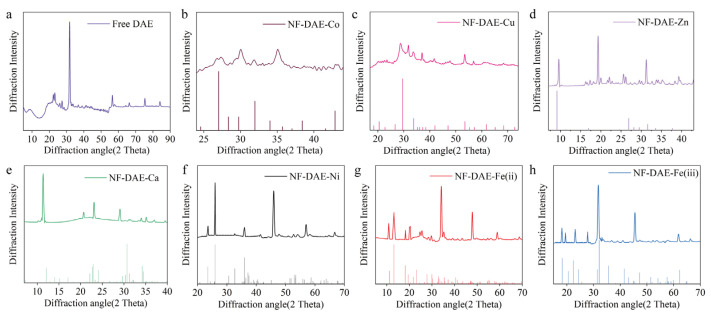
XRD analysis of free DAE and NF-DAEs. (**a**–**h**) show the XRD peaks of the free DAE, NF-DAE-Co, NF-DAE-Cu, NF-DAE-Zn, NF-DAE-Ca, NF-DAE-Ni, NF-DAE-Fe(ii), and NF-DAE-Fe(iii), respectively.

To verify the crystal formation of the NF-DAEs, the powders of the free DAE and these seven NF-DAEs were analyzed by XRD (Figure 3). The XRD peaks could be identified by comparing them with the standard card (CPDS, card 00-39-0702 for Co^2+^, card 00-22-0548 for Cu^2+^, card 00-39-0702 for Zn^2+^, card 00-029-0359 for Ca^2+^, card 00-038-1473 for Ni^2+^, card 00-029-0715 for Fe^2+^, and card 00-22-0548 for Fe^3+^, respectively). The NF-DAE-Co, NF-DAE-Cu, NF-DAE-Zn, NF-DAE-Ca, NF-DAE-Ni, NF-DAE-Fe(ii), and NF-DAE-Fe(iii) have fine peak structures, and most of the XRD peaks from the samples can be recognized as similar patterns to the standard card, which indicated that the NF-DAEs have good crystallinity.

Biomacromolecules and metal ions are essential for the formation of the biomimetic hybrid materials’ skeletons [29]. A hybrid nanomaterial is generally formed by the following three steps: first, the formation of the nucleus; second, the growth of metal nanostructures; third, the self-assembly of the metal nanostructures [30]. According to the SEM, seven metal ions formed nanoflower structures with DAE, which showed a high similarity to the repartitioned nanostructures of the corresponding metal ions [18,21,31]. The XRD and FTIR results further suggest that the formed nanostructure was a composite of protein and metal phosphate with a good crystallinity. However, the crystal structures formed by different metal ions and DAE are different, which may affect their catalytic activity and stability.

### 2.2. The Encapsulate Yields and Catalytic Activities of NF-DAEs

**Figure 4:**
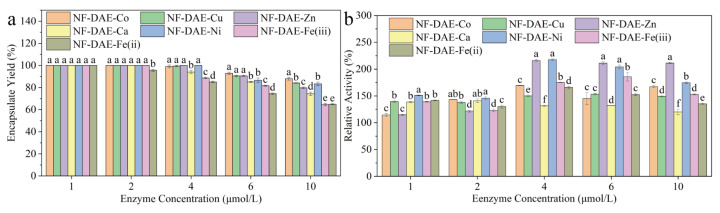
The encapsulate yield (**a**) and relative activities (**b**) of the NF-DAEs with different metal ions and DAE concentrations. The encapsulate yield was defined as the ratio of the immobilized DAE mass to the total DAE mass. The relative activity was defined as the ratio of the specific activity of the NF-DAEs to the activity of the free DAE. Different letters indicate significant differences at the *p* < 0.05 level. The initial free DAE activity was considered to be 100% of the relative activity.

To identify the capacity and activity of the NF-DAEs, we studied the impact of the enzyme-to-metal ion ratios on the encapsulate yields (Figure 4a) and catalytic activities (Figure 4b) by using different concentrations of DAE.

The encapsulation yield indicates the capacity of the NFs for protein immobilization. In our study, all of the NFs showed a high encapsulation yield (60~80%), even at a high enzyme concentration (10 μmol/L) and low mental ion concentration (1 mmol/L), indicating that DAE could form NFs with a high efficiency [32]. With low DAE concentrations of 1~2 μmol/L, most of the encapsulate yields were able to reach 100%, indicating the good immobilizing capacities of the NF-DAEs with all of the seven metal ions. However, with high DAE concentrations of 6~10 μmol/L, the NF-DAE-Co exhibited a high encapsulate yield, NF-DAE-Cu, NF-DAE-Zn, and NF-DAE-Ni exhibited lower encapsulate yields, and NF-DAE-Ca, NF-DAE-Fe(iii), and NF-DAE-Fe(ii) exhibited the lowest encapsulate yields (Figure 4a).

The present study has shown that the catalytic activities of the NF-DAEs were highly dependent on both the metal ions and the enzyme concentrations (Figure 4b). All of the NF-DAEs showed significantly higher catalytic activities than the free enzyme (Figure 4b), indicating an improvement in the activity when forming the NF-DAEs. The highest relative activity was achieved at 218% of the NF-DAE-Ni with the 4 μmol/L DAE. The NF-DAEs formed by Zn^2+^ and 4~10 μmol/L DAE showed relatively high activities (Figure 4b). Our results also show the significant increase in the catalytic activities of all of the NF-DAEs, which is consistent with other reports [17,26,33,34,35]. Therefore, in the subsequent preparation of the nanoflowers, the enzyme additions in NF-DAE-Co, NF-DAE-Cu, NF-DAE-Zn, NF-DAE-Ca, NF-DAE-Ni, NF-DAE-Fe(ii), and NF-DAE-Fe(iii) were all at a level of 4 μmol/L. The final enzyme amount was taken to be 1 μmol/L in the subsequent reaction of the NF-DAEs to catalyze the reaction.

The degree of increase in the catalytic activities of the NF-DAEs differed from the metal ions, which may be illustrated by the special structure of the nanoflowers in reducing the negative interactions between the DAE and metal nanostructures [36]. In a very recent study, Xin et al. reported the formation of NFs with DAE from and Cu^2+^, Co^2+^, Mn^2+^, and Zn^2+^ ions, and found that most of the resulted NFs decreased the catalytic activities, except for the NF-DAE-Co/Mn, indicating the performance of the NFs was closely related to the special enzyme immobilized [23].

### 2.3. Thermal Stabilities of NF-DAEs

**Figure 5:**
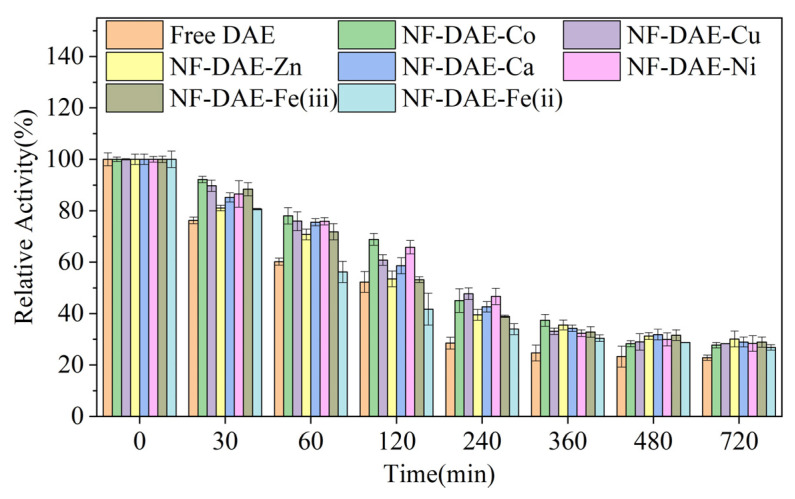
Thermal stabilities of the free DAE and NF-DAEs at 55 °C. Initial activity was considered to be 100% relative activity.

To evaluate the stabilities of the different NF-DAEs, we assayed the residual catalytic activities of the free DAE and NF-DAEs, which were incubated at 55 °C for 0~720 min (Figure 5). Along with the increase in the incubation time, the relative activity of the free DAE decreased sharply, whereas the relative activities of the NF-DAEs declined more slowly, indicating higher thermal stabilities for the NF-DAEs. Indeed, the half-lives of the NF-DAE-Co, NF-DAE-Cu, NF-DAE-Zn, NF-DAE-Ca, NF-DAE-Ni, and NF-DAE-Fe(iii) were 228, 174, 133, 170, 221, and 141 min, respectively, all of which were higher than that of the free DAE (105 min).

In the present study, most of the NF-DAEs improved the thermal stability of the enzyme, and similar results have been reported in previous studies [33,37]. This result may be related to the structure of the hybrid nanoflower, where the interaction between the organic and inorganic components provides a better secondary structure [38] that protects the immobilized enzyme at higher temperatures.

### 2.4. Co-Immobilization of NF-DAEs on Resins

#### 2.4.1. Preparation and Characterizations of Resin-NF-DAE Composites

**Figure 6:**
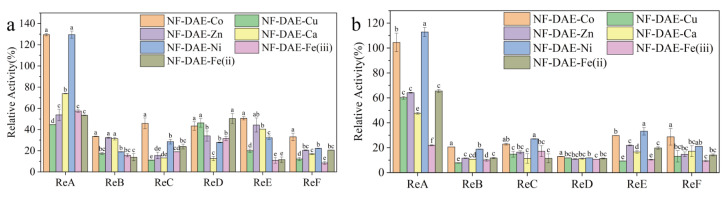
The relative activity of the resin-NF-DAE composites prepared by method A (**a**) and method B (**b**). ReA (LXTE-606 neutral hydrophobic epoxy-based polypropylene macroreticular resins), ReB (D202 macroprous strongly basic styrene-type anion-exchange resins), ReC (D113 weak acidic resins), ReD (LX-1000EP weak alkaline epoxy anion resins), ReE (AB-8 macroreticular adsorptive resins), and ReF (YKC101 strong acid cation-exchange resins). Method A: NF-DAEs were firstly prepared, and were then mixed with resins in PBS buffer and incubated at 30 °C for 4 h. Method B: resins were added to the solutions for the NF-DAE preparation at the beginning of the incubation step at 4 °C for 72 h. Different letters indicate significant differences at the *p* < 0.05 level. The initial free DAE activity was considered to be 100% of the relative activity.

Despite the NF-DAEs significantly increasing the catalytic activity and improving the thermal stability of DAE, we found those micrometer-sized particles were difficult to separate from the reaction solution (Figure S2, Additional File), especially when the density and viscosity were high (due to a high concentration of the substrate). Thus, the industrial application of NF-DAEs would be restricted due to the high energy cost and time cost in the separation step, and the gradual catalytic activity loses during the prolonged operation process as well. To solve this issue, we tried to prepare a composite material with immobilized NF-DAEs on different resins, including ReA (LXTE-606 neutral hydrophobic epoxy-based polypropylene macroreticular resins), ReB (D202 macroprous strongly basic styrene-type anion-exchange resins), ReC (D113 weak acidic resins), ReD (LX-1000EP weak alkaline epoxy anion resins), ReE (AB-8 macroreticular adsorptive resins), and ReF (YKC101 strong acid cation-exchange resins) by two methods. Method A: NF-DAEs were formed first, and then the resins were added. Method B: the resins were added during the formation of the nanoflowers. The relative activities of the composites formed by the NF-DAEs and the resins are shown in Figure 6. All of the composites exhibited catalytic activities, but the activity differed dramatically between the different resins and different NFs. In most cases, the enzyme activity of the composites formed by method A (Figure 6a) was higher than that of the composites formed by method B (Figure 6b), indicating that method A is more conducive to the maintenance of the catalytic activity of the NF-DAEs. Therefore, the properties of the composites prepared by method A are mainly presented in the subsequent experiments. Among the six types of resins, the composites formed by the ReA were more advantageous than the others. For the composites formed by the ReA, significant differences in the relative activity were observed between the NF-DAEs, and ReA-NF-DAE-Co and ReA-NF-DAE-Ni showed higher catalytic activities (up to 130%) compared to the other composites.

These differences in the enzyme activities of the composites may be due to the effects of the surface structure of the resins, the special nanoflower structures, and the interaction between the resins and nanoflowers. The higher catalytic activity of the ReA-NF-DAEs could be attributed to the fact that the macroporous resin is more favorable for binding with nanoflowers. Previously, lipase/Ca_3_(PO_4_)_2_ hybrid nano-immobilized on sulfonated macroporous resins at 60 °C showed a similar increase in activity compared to the free enzyme, suggesting that it is feasible to immobilize the nanoflowers on the resin [39].

#### 2.4.2. Characterization of ReA-NF-DAEs

**Figure 7:**
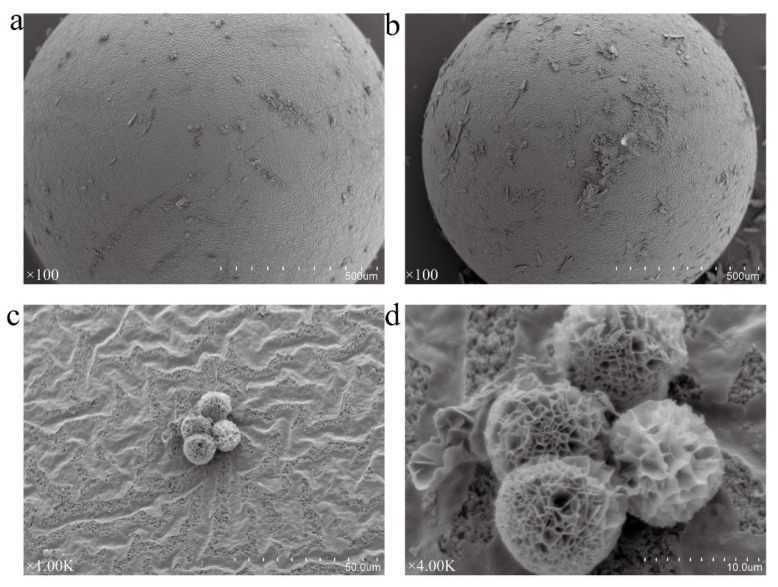
SEM images of ReA-NF-DAE-Co (**a**), ReA-NF-DAE-Ni (**b**), and partial images of the NF-DAE-Co structures on the ReA surface (**c**,**d**). Magnifications are in the lower-left corner of the image.

The ReA-NF-DAE-Co and ReA-NF-DAE-Ni prepared by method A (where NF-DAEs were firstly prepared, and were then mixed with resins in PBS buffer and incubated at 30 °C for 4 h) showed a high catalytic activity, so the structures of those two composites were further characterized by SEM (Figure 7). Both NF-DAE-Co and NF-DAE-Ni were adsorbed on the surface of the ReA to form a rough surface (Figure 7a,b). For ReA-NF-DAE-Co, flower-like spheres were clearly observed on the ReA surface (Figure 7a,c,d); whereas, for ReA-NF-DAE-Ni, nanosheets, instead of flower-like structures, were observed on the ReA surface (Figure 7b). ReA is a neutral hydrophobic epoxy-based polypropylene macroreticular resin, and showed a good adsorption performance to the epoxy group, indicating a strong adsorption effect to protein-based hybrid structures. Indeed, we observed that the NF-DAEs were adsorbed on the surface of the ReA (Figure 7a,b), which proved the interactions between the ReA and NF-DAEs. However, no nanostructures were observed in the holes of the ReA, which differs from a former report [39], and a possible reason is that the aperture size of the ReA is too small to load the nanoflowers. The NF-DAE-Co on the surface of the ReA (Figure 7d) showed a similar structure with the original nanoflower (Figure 1b), indicating that the adsorption of NF-DAE-Co on the resin surface did not destroy the morphology of the aggregation and crystallization of NF-DAE-Co. However, for NF-DAE-Ni, the original flower-like structure (Figure 1f) changed into nanosheets (Figure 7b), which may be related to the weak self-aggregation effect of DAE-Ni nanosheets, and the strong adsorption between the ReA and DAE-Ni nanosheets. Considering NF-DAE-Co and NF-DAE-Ni showed similar relative activity both before resin immobilization (Figure 4b) and after resin immobilization (Figure 6a), the destruction of the flower-like aggregation in ReA-NF-DAE-Ni would not lead to a reduction in the catalytic activity.

#### 2.4.3. Stabilities of ReA-NF-DAEs

**Figure 8:**
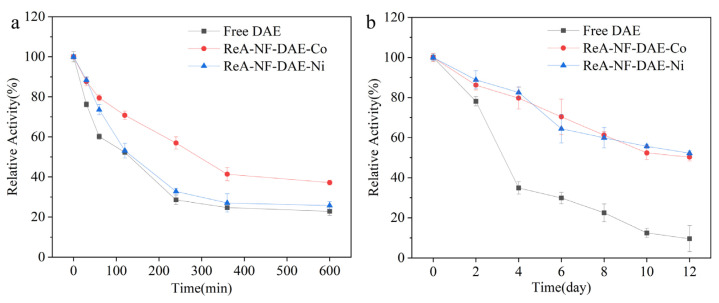
The thermal stability (**a**) and storage stability (**b**) of the free DAE and ReA-NF-DAEs. For thermal stability analysis, the samples were incubated at 55 °C for 0–720 min with continuous shaking. For storage stability analysis, the samples were stored at 4 °C and assayed for catalytic activity every two days. Initial activity was considered to be 100% relative activity.

The thermal and storage stabilities of immobilized enzymes play an important role on the potential industry applications. To evaluate the stabilities of different NF-DAEs, we assayed the residual catalytic activities of the free DAE and ReA-NF-DAEs, which were incubated at 55 °C for 0~720 min (Figure 8a). Along with the increase in the incubating time, the relative activity of the free DAE decreased sharply, whereas the relative activities of the ReA-NF-DAEs declined more slowly, indicating higher thermal stabilities for the ReA-NF-DAEs. Indeed, the half-lives of the ReA-NF-DAE-Co and ReA-NF-DAE-Ni samples were 221 and 142 min, respectively, which were higher than that of the free DAE (105 min).

To identify the storage stability of the ReA-NF-DAEs, we stored the free DAE and ReA-NF-DAEs at 4 °C for 2~12 days, and measured the residual catalytic activities (Figure 8b). The catalytic activity of the free enzyme decreased sharply with the prolongation of the storage time, whereas the catalytic activity of the ReA-NF-DAEs decreased more slowly, indicating that the ReA-NF-DAEs were more stable than the free DAE during storage (Figure 8b). The ReA-NF-DAEs maintained a catalytic activity above 50%, while the free DAE kept less than 10% of the initial activity after storage for 12 days.

Both the nanoflower-immobilized enzyme and the resin-immobilized enzyme were able to improve the enzyme stability [39]. Indeed, in our study, the formation of hybrid nanoflowers improved the thermal stability, and the half-life of the NF-DAE-Co reached 228 min, which is consistent with the half-life of the ReA-NF-DAE-Co (221 min). This suggests that the nanoflower structure dramatically influences the half-life of the enzyme in the resin–nanoflower composite structure.

#### 2.4.4. Processing Performances of ReA-NF-DAEs

**Figure 9:**
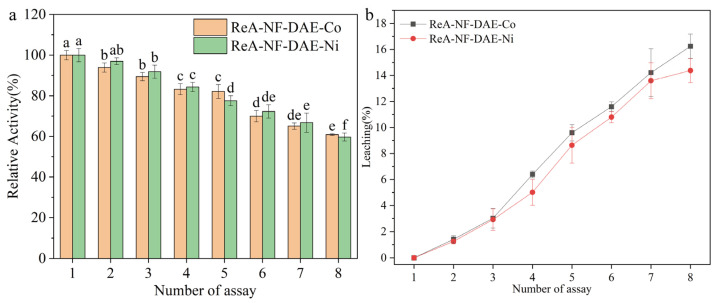
Operational stabilities of the ReA-NF-DAEs. (**a**) Relative activity. (**b**) Leaching of ReA-NF-DAE-Co and ReA-nf-dae-Ni. Reaction assays were taken at 55 °C for 1 h with 500 g/L D-fructose as the substrate. After a reaction, the ReA-NF-DAEs were simply pulled out from the mixture, and re-started another reaction cycle after being washed. Different letters indicate significant differences at the *p* < 0.05 level. Initial ReA-NF-DAE activity was considered to be 100% relative activity.

The processing performance is one of the key issues for industry application. Here, we evaluated the catalytic activities and leaching of ReA-NF-DAE-Co and ReA-NF-DAE-Ni for eight continuous reaction cycles (Figure 9). The residual activities of ReA-NF-DAE-Ni and ReA-NF-DAE-Co decreased slowly during repeated use (Figure 9a). After eight continuous reaction cycles, the ReA-NF-DAEs maintained 69.7% of the catalytic activity and 16.2% of the leaching (Figure 9b), indicating the good processing stability and processability of the ReA-NF-DAEs. Meanwhile, the residual activities of ReA-NF-DAE-Co and ReA-NF-DAE-Ni decreased after eight cycles, which may be related to the loss of the enzymes due to the leaching rate or the inactivation of the enzymes due to the high temperature.

Free DAE is difficult to reuse on an industrial scale, resulting in insufficient enzyme application and a waste in enzyme preparation. Although the formation of NFs can improve the catalytic activity and thermal stability of DAE, the high viscosity of the reaction solution in this experiment makes it difficult to recycle the NF-DAEs, even by using prolonged centrifugation or filtration for a long time, and leads to the continuous loss of catalytic activity during the operation. In our study, we combined the NF-DAEs with resins to form a composite material that not only enhances the catalytic activity and thermal and storage stability, but also enables easy recycling by simple depositing, filtering, or centrifugation. This would significantly reduce the enzyme consumption during the industrial D-allulose production.

## 3. Materials and Methods

### 3.1. Reagents and Materials

Ampicillin and Isopropyl β-D-1-Thiogalactopyranoside (IPTG) were purchased from Beijing Solarbio Science&Technology Co., Ltd. (Beijing, China). NaCl (99.5%), Na_2_HPO_4_·H_2_O (99%), NaH_2_PO_4_·H_2_O (99%), NiSO_4_·6H_2_O (99%), CoSO_4_·7H_2_O (99.5%), FeCl_3_·6H_2_O (99%), CaCl_2_·2H_2_O (99%), ZnSO_4_·7H_2_O (99.5%), CuSO_4_·5H_2_O (99%), and FeSO_4_·7H_2_O (99%) were purchased from Tianjin Bohai Chemical Reagent Co., Ltd. (Tianjin, China). D202 macroprous strongly basic styrene-type anion-exchange resins, LXTE-606 neutral hydrophobic epoxy-based polypropylene macroreticular resins, YKC101 strong acid cation-exchange resins, D113 weak acidic resins, AB-8 macroreticular adsorptive resins, and LX-1000EP weak alkaline epoxy anion resins were bought from Tianjin Yunkai Resin Technology Co., Ltd. (Tianjin, China).

### 3.2. Expression and Purification

A gene coding DAE from *Clostridium bolt* (GenBank: EDP19602.1, PDB:7X7W [40]) was synthesized (codon-optimized for *Escherichia coli*) and cloned into pET-22b between *Bam*HI and *Xho*I. The recombinant plasmid was transformed into *E. coli* BL21 (DE3) for DAE expression. The DAE expression and purification were performed according to the method described in the literature [41].

### 3.3. Activity Assays of DAE

The DAE activity was measured using 500 g/L D-fructose as the substrate. The enzymatic reaction was conducted in 50 mmol/L PBS buffer (pH 7.0) and 1.0 mmol/L Co^2+^. Free enzyme, NF, or resin-NF (with a final enzyme concentration of 1 μmo/L) were added to start the reaction, which was incubated at 55 °C for 20 min, followed by incubation at 100 °C for 10 min for the inactivation. The samples were then centrifuged at 13,000 r/min for 10 min, and the supernatants were filtered and diluted by 20-fold.

The concentration of D-allulose and D-fructose was detected using a high-performance liquid chromatography (HPLC) system equipped with a RID-20A refractive index detector (Shimadzu, Kyoto, Japan) and a Sugar-Pak I column (Waters, Milford, MA, USA) at 80 °C. The system was operated with deionized water as the mobile phase at a flow rate of 0.6 mL/min. The DAE activity was defined as the amount of enzyme required to generate 1 µmol D-allulose per minute at a pH of 7.0 and 55 °C [42].

### 3.4. Preparation of the Organic–Inorganic Hybrid Nanoflowers

The organic–inorganic hybrid nanoflowers were prepared according to the method described in the literature [16]. Briefly, CoSO_4_, CuSO_4_, CaCl_2_, NiSO_4_, ZnSO_4_, FeCl_3_, and FeSO_4_ were added to 1, 2, 4, 6, and 10 μmol/L of the DAE solution (in 50 mmol/L PBS buffer, pH of 7.0), respectively, to a final concentration of 1 mmol/L. The mixtures were placed at 4 °C for 72 h to form 7 NF-DAEs with different metal ions (named NF-DAE-Co, NF-DAE-Cu, NF-DAE-Zn, NF-DAE-Ca, NF-DAE-Ni, NF-DAE-Fe(iii), and NF-DAE-Fe(ii), respectively). The precipitates were washed twice with PBS buffer and collected by centrifugation at 13,000 r/min for 20 min. 

### 3.5. Assay of Encapsulation Yield

Encapsulation yield is defined as the immobilization efficiency of the enzyme. To calculate the encapsulation yield, a mixture of DAE, PBS, and metal ions was placed for 72 h and the concentration of the DAE in the supernatant was determined using the BCA protein concentration assay kit. The encapsulation yield was determined by Equation (1), as follows:

(1) $$EY(\%)=\frac{C_{0}-C_{1}}{C_{0}}\times 100$$

EY: the encapsulate yield;
C_0_: the total DAE mass;
C_1_: the supernatant DAE mass.

### 3.6. Characterization of the NF-DAEs and Re-NF-DAEs

The morphologies and structures of the NF-DAEs and Re-NF-DAEs were characterized with a Scanning Electron Microscope (SEM, SU1510, Hitachi, Tokyo, Japan). To characterize the Fourier transform infrared (FTIR, IS50, Thermo Fisher Scientific, Waltham, MA. USA) spectra of the NF-DAEs, the samples were pressed to form a tablet with KBr and were scanned at a range of 500~4000 cm^−1^ wave numbers by using an FTIR spectrometer. The crystal structures of the NF-DAEs were studied using X-ray diffraction (XRD, 6100, Shimadzu, Kyoto, Japan) with Cu Kα radiation.

### 3.7. Synthesis of Resin-Immobilized NF-DAE Composites

Composites of resin-immobilized NF-DAEs (Re-NF-DAEs) were prepared to improve the stability of the NF-DAEs by two different methods using seven resins and six NF-DAEs, respectively. Method A: NF-DAEs were firstly prepared as described in Section 3.4; NF-DAEs were then mixed with 6 resins in PBS buffer, respectively, and incubated at 30 °C for 4 h with shaking. Method B: the 6 resins were added to the solutions for the NF-DAE preparation (Section 3.4) at the beginning of the incubation step at 4 °C for 72 h, respectively, to form the Re-NF-DAEs directly. Afterward, the Re-NF-DAEs were washed with PBS buffer 2 times to remove the unreacted DAE.

### 3.8. Thermal Stability of the free DAE, NF-DAEs and Re-NF-DAEs

To characterize the thermal stability, the free DAE, NF-DAEs, or Re-NF-DAEs were incubated in PBS buffer at 55 °C for 0~720 min with continuous shaking, and then the residual activities were measured and used to calculate half-life time.

### 3.9. Storage Stability of the Re-NF-DAEs

The free DAE and Re-NF-DAEs were stored at 4 °C, and the residual activity was detected every two days.

### 3.10. Operational Stabilities of the Re-NF-DAEs

To characterize the processability, the Re-NF-DAEs were used to catalyze the D-allulose conversion (Re-NF-DAEs were added to the reaction regent with 50 mmol/L PBS, 1 mmol/L Co^2+^, 500 g/L D-fructose as substrate, and incubated at 55 °C for 1 h) for 8 repeated cycles. The Re-NF-DAEs were recycled by being pulled out from the reaction regent after each conversion, washed with PBS buffer twice, and then used for the next conversion. The residual activities of the Re-NF-DAEs were analyzed by using samples of the reaction for 20 min through HPLC. 

The leaching of the Re-NF-DAEs were determined by Equation (2), as follows:

(2) $$\mathrm{Leaching}\;(\%)=\frac{A_{1}}{A_{0}}$$

A_0_: the amount of enzyme in the initial immobilized enzyme;
A_1_: the accumulated amount of enzyme in the supernatant after the reaction.

### 3.11. Data Analysis

We carried out at least three experiments for each test. Data were analyzed using SPSS version 15 software by the one-way Analysis of Variance (ANOVA) method with LSD, Tukey’s s-b, and Duncan’s test. Statistical significance was defined as *p* < 0.05. All experimental values were presented as mean values ± standard deviations.

## 4. Conclusions

In this work, organic–inorganic hybrid nanoflowers (NFs) were formed by the combination of D-allulose 3-epimerase (DAE) and seven different metal ions, among which NF-DAE-Ni showed the highest relative activity and thermal stability. We also demonstrated the formation of resin-NF composites, which showed higher catalytic activity and thermal and storage stability, and which could be easily recycled from the reaction solution compared with the free DAE. The composite ReA-NF-DAE-Co retained more than 60% of its DAE activity after eight reaction cycles. Our results demonstrate a novel strategy combining NFs and resin immobilization for a highly efficient, stable, and reusable enzymic reaction, not only for D-allulose production, but also for similar enzymic processes.

## Data Availability

The data supporting the conclusions of this article are listed in the text.

## Supplementary Materials

The following supporting information can be downloaded at: https://www.mdpi.com/article/10.3390/ijms25126361/s1.

## References

[1] Mahmood S., Iqbal M.W., Tang X., Zabed H.M., Chen Z., Zhang C., Ravikumar Y., Zhao M., Qi X. (2024). A comprehensive review of recent advances in the characterization of L-rhamnose isomerase for the biocatalytic production of D-allose from D-allulose. Int. J. Biol. Macromol..

[2] Hossain A., Yamaguchi F., Matsuo T., Tsukamoto I., Toyoda Y., Ogawa M., Nagata Y., Tokuda M. (2015). Rare sugar D-allulose: Potential role and therapeutic monitoring in maintaining obesity and type 2 diabetes mellitus. Pharmacol. Ther..

[3] Zhang W., Chen D., Chen J., Xu W., Chen Q., Wu H., Guang C., Mu W. (2023). D-allulose, a versatile rare sugar: Recent biotechnological advances and challenges. Crit. Rev. Food Sci. Nutr..

[4] Bae H.R., Shin S.K., Han Y., Yoo J.H., Kim S., Young H.A., Kwon E.Y. (2023). D-Allulose Ameliorates Dysregulated Macrophage Function and Mitochondrial NADH Homeostasis, Mitigating Obesity-Induced Insulin Resistance. Nutrients.

[5] Rakhat Y., Kaneko K., Wang L., Han W., Seino Y., Yabe D., Yada T. (2022). D-Allulose Inhibits Ghrelin-Responsive, Glucose-Sensitive and Neuropeptide Y Neurons in the Arcuate Nucleus and Central Injection Suppresses Appetite-Associated Food Intake in Mice. Nutrients.

[6] Su L., Sun F., Liu Z., Zhang K., Wu J. (2018). Highly efficient production of Clostridium cellulolyticum H10 D-psicose 3-epimerase in Bacillus subtilis and use of these cells to produce D-psicose. Microb. Cell Fact..

[7] Dedania S.R., Patel M.J., Patel D.M., Akhani R.C., Patel D.H. (2017). Immobilization on graphene oxide improves the thermal stability and bioconversion efficiency of D-psicose 3-epimerase for rare sugar production. Enzym. Microb. Technol..

[8] Gao X., Wei C., Qi H., Li C., Lu F., Qin H.M. (2023). Directional immobilization of D-allulose 3-epimerase using SpyTag/SpyCatcher strategy as a robust biocatalyst for synthesizing D-allulose. Food Chem..

[9] He W., Jiang B., Mu W., Zhang T. (2016). Production of D-allulose with D-psicose 3-epimerase expressed and displayed on the surface of Bacillus subtilis spores. J. Agric. Food Chem..

[10] Xue K., Liu C.L., Yang Y., Liu X., Zhan J., Bai Z. (2022). Immobilization of D-allulose 3-epimerase into magnetic metal-organic framework nanoparticles for efficient biocatalysis. World J. Microbiol. Biotechnol..

[11] Gong C., Wang D., Zhao H. (2023). Biomimetic Metal-Pyrimidine Nanoflowers: Enzyme Immobilization Platforms with Boosted Activity. Small.

[12] Patil P.D., Kelkar R.K., Patil N.P., Pise P.V., Patil S.P., Patil A.S., Kulkarni N.S., Tiwari M.S., Phirke A.N., Nadar S.S. (2023). Magnetic nanoflowers: A hybrid platform for enzyme immobilization. Crit. Rev. Biotechnol..

[13] Xu K., Appiah B., Zhang B., Yang Z., Quan C. (2023). Recent advances in enzyme immobilization based on nanoflowers. J. Catal..

[14] Al-Maqdi Khadega A., Elmerhi N., Alzamly A., Shah I., Ashraf S. (2023). Laccase–copper phosphate hybrid nanoflower as potent thiazole remediation agent. J. Water Process.

[15] Mostafavi M., Mahmoodzadeh K., Habibi Z., Yousefi M., Brask J., Mohammadi M. (2023). Immobilization of Bacillus amyloliquefaciens protease "Neutrase" as hybrid enzyme inorganic nanoflower particles: A new biocatalyst for aldol-type and multicomponent reactions. Int. J. Biol. Macromol..

[16] Ge J., Lei J., Zare R.N. (2012). Protein-inorganic hybrid nanoflowers. Nat. Nanotechnol..

[17] Mostafavi M., Poor M.B., Habibi Z., Mohammadi M., Yousefi M. (2024). Hyperactivation of lipases by immobilization on superhydrophobic graphene quantum dots inorganic hybrid nanoflower. Int. J. Biol. Macromol..

[18] Kiani M., Mojtabavi S., Jafari-Nodoushan H., Tabib S.-R., Hassannejad N., Faramarzi Mohammad A. (2022). Fast anisotropic growth of the biomineralized zinc phosphate nanocrystals for a facile and instant construction of laccase@ Zn_3_(PO_4_)_2_ hybrid nanoflowers. Int. J. Biol. Macromol..

[19] Fu L., Yao Y., Ma J., Zhang Z., Wang G., Wei W. (2024). Nanoflower-like NiCo_2_O_4_ Composite Graphene Oxide as a Bifunctional Catalyst for Zinc–Air Battery Cathode. Langmuir.

[20] Tu X., Sun Q., Zhu S., Sun C., Qu J., Zhu Z., Zhang D., Zheng H. (2024). Nanoflower Fe-base complex for efficient CO_2_ fixation under atmospheric pressure. J. Environ..

[21] Moya C., Escoda-Torroella M., Rodríguez-Álvarez J., Figueroa A., García Í., FerrerVidal Inés B., GalloCordova A., Puerto Morales M., Aballe L., Fraile Rodríguez A. (2024). Unveiling the crystal and magnetic texture of iron oxide nanoflowers. Nanoscale.

[22] Ocsoy I., Dogru E., Usta S. (2015). A new generation of flowerlike horseradish peroxides as a nanobiocatalyst for superior enzymatic activity. Enzyme Microb. Technol..

[23] Gao X., Fang S., Ma X., Wang T., Li C., Lu F., Qin H. (2024). Customized self-assembled bimetallic hybrid nanoflowers promoting the robustness of D-allulose 3-epimerase. Chem. Eng. J..

[24] Patel S.K.S., Choi H., Lee J.K. (2019). Multimetal-based inorganic–protein hybrid system for enzyme immobilization. Acs Sustain. Chem. Eng..

[25] Patel S.K.S., Gupta R.K., Karuppanan K.K., Padhi D.K., Ranganathan S., Paramanantham P., Lee J.K. (2024). Trametes versicolor Laccase-Based Magnetic Inorganic-Protein Hybrid Nanobiocatalyst for Efficient Decolorization of Dyes in the Presence of Inhibitors. Materials.

[26] Xiang X., Xiong Y., Zhang Q., Lei H., Liu K., Wang S. (2022). Ionic liquids modified Cu_3_(PO_4_)_2_ hybrid nanoflower for dehydrogenase immobilization by biomimetic mineralization. Process Biochem..

[27] Hartmann M., Kostrov X. (2013). Immobilization of enzymes on porous silicas–benefits and challenges. Chem. Soc. Rev..

[28] Cheng S., Guo Z., Liang C., Shi Y., Geng P., Xin Y., Gu Z., Zhang L. (2021). Immobilization of Phospholipase A1 Using a Protein-Inorganic Hybrid System. Polymers.

[29] Xu H., Liang H. (2022). Chitosan-regulated biomimetic hybrid nanoflower for efficiently immobilizing enzymes to enhance stability and by-product tolerance. Int. J. Biol. Macromol..

[30] Liu D., Chen Z., Long J., Zhao Y., Du X. (2018). Immobilization of Penicillin Acylase on Macroporous Adsorption Resin CLX 1180 Carrier. Adv. Polym. Tech..

[31] Zhang X., Li B., Lan M., Yang S., Xie Q., Xiao J., Xiao F., Wang S. (2021). Cation modulation of cobalt sulfide supported by mesopore-rich hydrangea-like carbon nanoflower for oxygen electrocatalysis. ACS Appl. Mater..

[32] Zhu H., Chen J., Zhang Y., Goh K.L., Wan C., Zheng D., Zheng M. (2023). Preparation and investigation of novel endopeptidase-exopeptidase co-immobilized nanoflowers with improved cascade hydrolysis. Int. J. Biol. Macromol..

[33] Li Y., Yan L., Liu G., Chen H., Zhao H., Wang L., Gao J., Liu Y., Zheng X., Jiang Y. (2023). Enhanced electroenzymatic CO_2_ reduction by a multifunctional ZIF-8 layer on silica nanoflower with immobilized enzyme. Chem. Eng. J..

[34] Ma X., Chen Z., Han J., Zhou Y., Lin F., Li C., Wang L., Wang Y. (2022). Fabrication of immobilized bromelain using cobalt phosphate material prepared in deep eutectic solvent as carrier. Colloids Surf. B Biointerfaces.

[35] Badoei-Dalfard A., Monemi F., Hassanshahian M. (2022). One-pot synthesis and biochemical characterization of a magnetic collagenase nanoflower and evaluation of its biotechnological applications. Colloids Surf. B Biointerfaces.

[36] Cui J., Zhao Y., Liu R., Zhong C., Jia S. (2016). Surfactant-activated lipase hybrid nanoflowers with enhanced enzymatic performance. Sci. Rep..

[37] Wang Z., Liu P., Fang Z., Jiang H. (2022). Trypsin/Zn_3_(PO_4_)_2_ Hybrid Nanoflowers: Controlled Synthesis and Excellent Performance as an Immobilized Enzyme. Int. J. Mol. Sci..

[38] Wang D., Hu L., Xu R., Zhang W., Xiong H., Wang Y., Du G., Kang Z. (2023). Production of different molecular weight glycosaminoglycans with microbial cell factories. Enzym. Microb. Technol..

[39] Wan D., Tian L., Li X., Li B., Zhang Q. (2018). A versatile strategy for enzyme immobilization: Fabricating lipase/inorganic hybrid nanostructures on macroporous resins with enhanced catalytic properties. Biochem. Eng. J..

[40] Xie X., Tian Y., Ban X., Li C., Yang H., Li Z. (2022). Crystal structure of a novel homodimeric D-allulose 3-epimerase from a Clostridia bacterium. Acta Crystallogr. D.

[41] Qi H., Wang T., Li H., Li C., Guan L., Liu W., Wang J., Lu F., Mao S., Qin H.M. (2023). Sequence- and Structure-Based Mining of Thermostable D-Allulose 3-Epimerase and Computer-Guided Protein Engineering To Improve Enzyme Activity. J. Agric. Food Chem..

[42] Patel S.N., Kaushal G., Singh S.P. (2020). A Novel d-Allulose 3-Epimerase Gene from the Metagenome of a Thermal Aquatic Habitat and d-Allulose Production by Bacillus subtilis Whole-Cell Catalysis. Appl. Environ. Microbiol..

