# Supplementary material for: The Formation of D-Allulose 3-Epimerase Hybrid Nanoflowers and Co-Immobilization on Resins for Improved Enzyme Activity, Stability, and Processability
Source: Int J Mol Sci. 2024 Jun 8;25(12):6361. doi: 10.3390/ijms25126361 (PMC11203923; doi:10.3390/ijms25126361)
Supplement: Supplementary file 1 [file ijms-25-06361-s001.zip › ijms-3038980-supplementary.pdf]

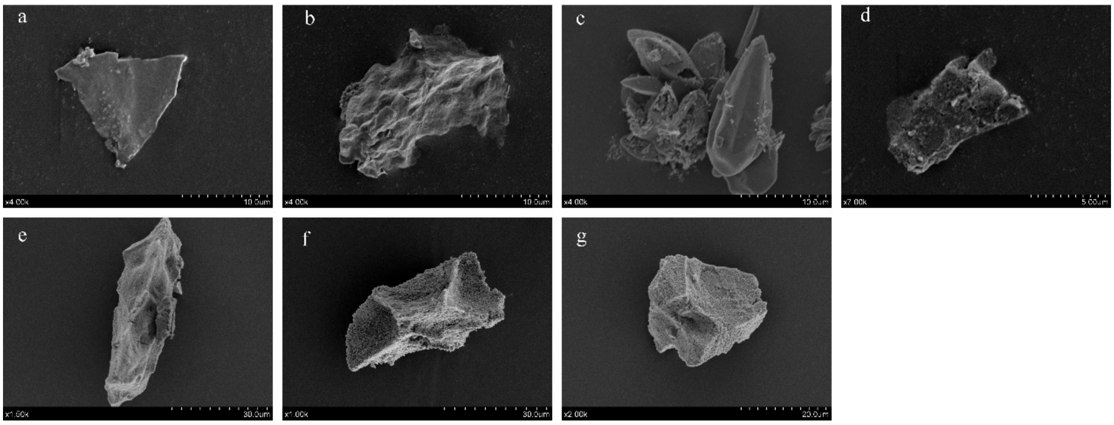

Figure S1. SEM images of metal phosphate. (a) $Co_3(PO_4)_2$, (b) $Cu_3(PO_4)_2$, (c) $Zn_3(PO_4)_2$, (d) $Ca_3(PO_4)_2$, (e) $Ni_3(PO_4)_2$, (f) $Fe_3(PO_4)_2$, and (g) $FePO_4$. Magnifications are marked in the lower left corner of the image.

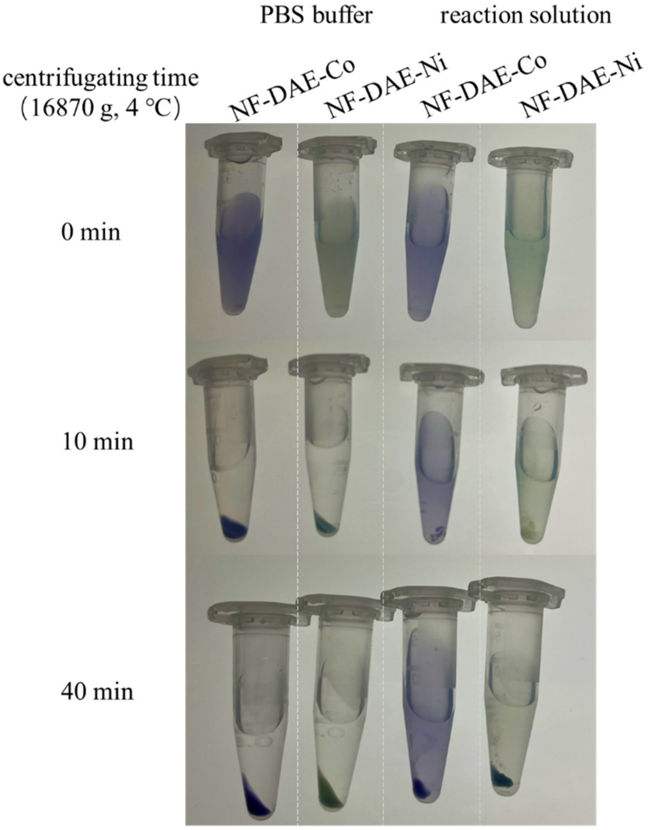

Figure S2. Nanoflowers after centrifugation in PBS buffer and reaction solution, respectively.
